# Antihydropin

**Published:** 1877-05

**Authors:** 


ANTIHYDROPIN.—This is the active principle of the cockroach (blatta orientalis), and is said to be a favorite remedy of the Russians, for dropsical affusions from disease of the kidneys, heart, or other organs.

The powdered cockroach is used in substance, five to ten grains in twenty-four hours, and, unlike cantharades, has no irritating effect upon the urinary organs. The urine and perspiration is said to be greatly increased, and if albumen be present, soon disappears from the secretion.

These facts are gleaned from the *Library*, on the responsibility of an exchange.

